# The Evolution of Risk Classification for Neuroblastoma

**DOI:** 10.3390/children6020027

**Published:** 2019-02-11

**Authors:** Elizabeth Sokol, Ami V. Desai

**Affiliations:** 1 Division of Hematology, Oncology, Neuro-Oncology and Stem Cell Transplant, Ann & Robert H. Lurie Children’s Hospital of Chicago, Chicago, IL 60611, USA; esokol@luriechildrens.org; 2 Department of Pediatrics, Section of Hematology, Oncology and Stem Cell Transplantation, The University of Chicago, Chicago, IL 60637, USA; 3 Committee on Clinical Pharmacology and Pharmacogenomics, The University of Chicago, Chicago, IL 60637, USA

**Keywords:** neuroblastoma, risk classification

## Abstract

Neuroblastoma is a tumor with great clinical heterogeneity. Patients in North America are risk-stratified using a number of features including age at diagnosis, disease stage, tumor histology, *MYCN* status (amplified versus nonamplified), and tumor cell ploidy. In this paper, we review the evidence for utilizing these features in the risk classification of neuroblastic tumors. Additionally, we review the clinical and biologic criteria used by various cooperative groups to define low, intermediate, and high-risk disease populations in clinical trials, highlighting the differences in risk classification internationally. Finally, we discuss the development of the International Neuroblastoma Risk Group classification system, designed to begin worldwide standardization of neuroblastoma pretreatment risk classification and allow comparison of clinical trials conducted through different cooperative groups.

## 1. Introduction

**Figure 1:**
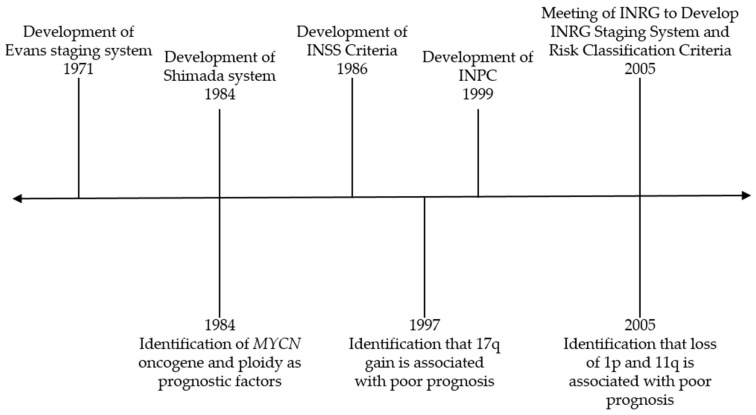
Timeline of major events contributing to risk classification for neuroblastoma. INSS—International Neuroblastoma Staging System; INPC—International Neuroblastoma Pathology Classification; INRG—International Neuroblastoma Risk Group.

One of the hallmarks of neuroblastoma is its clinical heterogeneity. While children with low-risk disease may be observed or undergo surgery and those with intermediate-risk disease may receive chemotherapy and undergo surgical resection, those with high-risk disease receive intensive, multimodality therapy that includes chemotherapy, surgery, myeloablative chemotherapy with autologous stem cell rescue, radiation, and immunotherapy with an anti-GD2 antibody [1,2,3]. Thus, it is critical to appropriately risk-stratify patients to ensure that they receive the optimal treatment regimen. Over the last several decades, numerous clinical and biologic factors have been incorporated into the risk classification system for neuroblastoma to categorize patients as having low, intermediate, or high-risk disease. Figure 1 illustrates major events contributing to modern risk classification and will be discussed in this review. Efforts have been made to uniformly define risk groups across cooperative groups through the International Neuroblastoma Risk Group (INRG) classification system [4]. 

## 2. Risk Classification: Key Clinical and Biologic Factors

The Children’s Oncology Group (COG) has traditionally used the following factors to stratify patient risk. (1) Age at diagnosis, (2) stage to define extent of disease by International Neuroblastoma Staging System (INSS), (3) tumor histology using International Neuroblastoma Pathology Classification (INPC) criteria, (4) *MYCN* status, and (5) DNA index or tumor cell ploidy. 

### 2.1. Age at Diagnosis

Older age has been prognostic of poor outcome in neuroblastoma since the 1970s. Earlier studies demonstrated that patients over 12 months of age at diagnosis had inferior outcomes [5]. Upon retrospective review of 3666 patients treated on Pediatric Oncology Group (POG) or Children’s Cancer Group (CCG) studies (POG and CCG being predecessors to the COG), London et al. [6] demonstrated that the prognostic significance of age on outcome is continuous in nature and suggested that an age cutoff between 12 and 18 months, rather than 365 days, could be utilized for clinical risk stratification. The CCG and POG groups demonstrated favorable prognosis in children 12–18 months of age with metastatic, *MYCN* nonamplified disease and children with metastatic, *MYCN* nonamplified, hyperdiploid disease, compared to older children thus supporting a prognostic age cut-off of 18 months and potentially less intensive therapy for those 12–18 months with other favorable prognostic markers [7,8]. Metastatic disease in children under 18 months of age is not associated with the poor outcomes that result in older patients.

During development of the INRG risk classification system (detailed later in this review), an analysis of non-COG patients in the INRG cohort supported an age cutoff between 15 and 19 months. While it was again acknowledged that the prognostic significance of age is continuous, an age cutoff of 18 months (547 days) was chosen by the INRG task force for clinical purposes. However, for a subgroup of patients with diploid, *MYCN* nonamplified tumors and distant metastatic disease, the INRG task force recommended an age cutoff of 12 months (365 days) for the INRG classification system [4]. Additional analyses showed that age retained prognostic significance in more modern cohorts treated with intensified therapy and supported an older age cutoff of greater than 18 months at diagnosis as a risk criterion [4,9]. 

### 2.2. Disease Stage

In 1971, Evans et al. [10] published the Evans Staging System based on extent of disease including a IV-S category, recognizing that there is a cohort of patients with metastatic disease limited to the skin, liver, and bone marrow, who have superior outcomes. Subsequently in 1986, an international group convened to develop a surgical staging system to aid in comparison of outcomes and therapies between countries, as various staging systems were being used worldwide [11]. The International Neuroblastoma Staging System (INSS) was developed taking into account degree of tumor resection, presence of ipsilateral or contralateral lymph nodes involvement, tumor infiltration across the midline of the body, and separation of patients with INSS stage 4S (infants with specific metastatic disease pattern including only liver, skin, and bone marrow) from other children with metastatic disease (INSS stage 4) to harmonize staging across groups, see Table 1 for definitions of stages [11]. Survival among patients with INSS stage 4 disease was significantly worse than those with stages 1, 2, 3, or 4S disease [12]. Staging was again addressed in 2005 during a meeting of the International Neuroblastoma Risk Group (INRG) task force and a system utilizing image-defined risk factors in place of degree of surgical resection was developed (INRG Staging System, or INRGSS) [4,13,14]. This system would allow for pretreatment staging rather than postsurgical staging. This is important in patients with localized disease who do not require surgical resection such as those with perinatally diagnosed disease. These patients could not be properly staged with INSS as surgical resection is required to define stage 1 or 2 disease. European groups had already adopted the image-defined risk factors to eliminate surgical style or skill from the evaluation of risk. In addition to the use of radiographic features in place of surgical resection, several other changes were included in the INRG system including elimination of lymph node assessment and midline nature of tumors and use of 18 months instead of 12 months to define MS disease. The INRGSS (Table 2) is now being incorporated into new protocols developed through the Children’s Oncology Group. 

The methods used to perform the staging evaluation have changed over time as well. Bone scans were previously used to asses for metastatic disease to the bone. This has been replaced by the use of I-123-metaiodobenzylguanidine (I-123-MIBG) scans [2]. I-123-MIBG is a radiotracer that is taken up by the norepinephrine transporter. The majority of neuroblastoma cells take up this tracer, allowing for a more specific mechanism to detect metastatic disease. In patients with MIBG non-avid tumors, fluorodexoyglucose (FDG)-positron emission topography (PET) is used. CT and/or MRI is used to evaluate for soft tissue disease. Bilateral bone marrow aspirate and biopsy is used to evaluate for the presence of metastatic disease in the bone marrow. 

### 2.3. Tumor Histology

Shimada et al. [15] developed the first histology grading system in 1984 to classify neuroblastic tumors by histologic features. Features including degree of stroma present, grade of differentiation, mitosis-karyorrhexis index (MKI) [16], presence of nodules, and age were utilized to define groups with either favorable or unfavorable prognosis. The International Neuroblastoma Pathology Classification (INPC) was then developed in 1999 to update these histologic factors impacting prognosis [17,18]. INPC incorporates multiple factors consisting of diagnostic category (accounting for quantity of Schwannian stromal development and grade of tumor differentiation), MKI, and age to ultimately define tumor histology as favorable versus unfavorable (Table 3). Diagnostic categories include ganglioneuroma (Schwannian stroma-dominant) with mature or maturing subtypes; ganglioneuroblastoma, intermixed (Schwannian stroma-rich); ganglioneuroblastoma, nodular (composite Schwannian stroma-rich/stroma-dominant and stroma-poor); and neuroblastoma (Schwannian stroma-poor) with undifferentiated, poorly differentiated, and differentiating subtypes. This provides greater subdivision than the Shimada classification that only defined stroma-poor and stroma-rich. Grade was further subdivided as well to include three categories (differentiating, poorly differentiated, and undifferentiated) instead of the two included in the Shimada system (differentiating and undifferentiated). MKI reflects the degree of cell replication seen in a high-power microscope field and categorized as low (<2% or <100/5000 mitotic and karyorrhectic cells), intermediate (<2%–4% or <100–200/5000 mitotic and karyorrhectic cells), and high (>4% or >200/5000 mitotic and karyorrhectic cells). Age is not incorporated into prognostic grouping for ganglioneuroblastoma intermixed and ganglioneuroma, which fall within the favorable histology group or ganglioneuroblastoma, nodular which is considered an unfavorable histology. Age and MKI, however, impact prognostic grouping within tumors categorized as neuroblastoma. Neuroblastoma tumors with favorable histology follow a framework of age-linked maturation and include poorly differentiated (age <1.5 years) to differentiating (age <5 years) neuroblastoma and should have low (age <5 years) or up to intermediate (age <1.5 years) MKI. In contrast, tumors with unfavorable histology demonstrate features that suggest aggressive growth and have immature histologies for the age of the patient. Within neuroblastoma tumors, the unfavorable histology group includes undifferentiated histology (in any age) or poorly differentiated subtype (age ≥1.5 years) or any subtype (age ≥5 years). Further, those with high MKI (in any age) or intermediate (age ≥1.5 years) qualify as having unfavorable histology [17]. Age is included in INPC and contributes to the prognostic significance of the histologic groups. Thus, within the risk classification system, age is weighted twice—both within INPC and independently. 

### 2.4. MYCN Status

Several genetic factors predictive of outcome in neuroblastoma have been identified, beginning in the 1980s with *MYCN* (or *N-myc*) status and DNA index, or tumor cell ploidy. *MYCN* is an oncogene located on the short arm of chromosome 2. Amplification of *MYCN* was identified in neuroblastoma cell lines and then in untreated tumors. Brodeur et al. [19] demonstrated an association between amplification of *MYCN* and higher stage tumors in early analyses. Seeger et al. [20] identified that *MYCN* amplification was associated with shorter progression free survival in all stages of disease. *MYCN* was the first clinically relevant genetic biomarker in cancer [21]. Approximately 20% of primary neuroblastoma tumors demonstrate *MYCN* amplification. Biologically, *MYCN* is involved in many processes leading to aggressive disease including migration/metastases, cell survival, apoptosis, proliferation, pluripotency, self-renewal, angiogenesis, and blocking cell cycle arrest, immune surveillance, and differentiation [22]. *MYCN* amplification remains one of the strongest predictors of high-risk disease [23]. Currently, COG uses fluorescence in situ hybridization to determine *MYCN* status [24]. Samples with a 4-fold increase in signal compared to a centromeric reference are considered amplified. Those with 2–3-fold increase have *MYCN* gain. Other methods can be used to determine amplification as well including polymerase chain reaction, array-based comparative genomic hybridization, and multiplex ligation-dependent probe amplification [25].

### 2.5. Tumor Cell Ploidy

Tumor ploidy was identified as a prognostic factor around the time the role of *MYCN* amplification in neuroblastoma was identified. Tumors with DNA index less than or equal to 1 have poorer outcomes than hyperdiploid tumors [26,27]. In 1984, Look et al. [27] showed that higher DNA index was associated with better response to therapy in infants with unresectable tumors. Kaneko et al. then showed the association between near triploid tumors and favorable disease. Diploid and near-tetraploid tumors were associated with more aggressive disease [28]. Patients between 12 and 18 months with *MYCN* nonamplified, hyperdiploid tumors were found to have superior outcomes than the same group with diploid tumors [8]. 

### 2.6. Chromosomal Aberrations

Although *MYCN* and tumor cell ploidy are the two biologic markers that have been classically utilized by the COG to assign risk groups, chromosomal aberrations have been identified and used by different groups. Certain segmental chromosome aberrations were found to be recurrent and associated individually with inferior outcomes. Loss of heterozygosity at 1p and 11q and 17q gain have been found to be predictive of poorer survival in patients with neuroblastoma [29,30]. COG utilized 1p and 11q status for treatment assignment on the intermediate risk study ANBL0531 (NCT NCT00499616), such that loss of heterozygosity (LOH) at 1p36 and/or 11q23 precluded patients from receiving therapy reduction [31]. Because 1p loss and 17q gain are associated with *MYCN* amplification—but 11q loss is not—11q aberration was incorporated into the INRG risk classification as a predictor of poor outcome that is independent of *MYCN* status [4,29,30]. 

The presence of whole versus partial chromosomal aberrations has also been found to correlate with clinical behavior of the tumor. Schleiermacher and colleagues evaluated types of chromosomal changes and their impact on outcomes in patients with *MYCN* nonamplified tumors [32]. They identified that patients with whole chromosome gains and losses had significantly better outcomes than patients with partial or segmental chromosome aberrations. The presence of segmental chromosome aberrations has been incorporated as an unfavorable genomic feature in COG ANBL1232 (NCT02176967), an ongoing study that is using response and biologic criteria to decrease therapy in a select cohort with localized non-high-risk disease. A favorable genomic profile, including absence of any segmental chromosome aberration (1p, 3p, 4p, or 11q loss or 1q, 2p, or 17q gain) and one or more whole chromosome gains, and favorable histology allow for observation, rather than initiating chemotherapy, for patients with L2 tumors. The ability to observe patients with large tumors with favorable biology remains a study question. 

### 2.7. Other Lab Findings

Other labs including ferritin and lactic dehydrogenase (LDH) have been found to prognostic as well. Elevated ferritin has been found to be correlated with worse outcome in patients with Evans stage III and IV disease [33]. It is also elevated more often in patients with Evans stage IV disease in comparison to those with stage IV-S disease [34]. Elevated LDH has similarly been correlated with poorer prognosis [35]. These markers were some of the earliest indicators of poor prognosis. Currently, these markers are not routinely used in evaluation of patients with neuroblastoma as they are not specific, but they may be useful in combination with other more specific prognostic features.

## 3. Variation in Risk Classification Among Cooperative Groups and the Development of the INRG Risk Classification System

Cooperative groups have used different sets of prognostic factors to define risk groups, making it difficult to compare treatments between studies. Several studies have been completed around the world in patients with low, intermediate, and high-risk disease using various inclusion criteria (Table 4). Between 1998 and 2004, the COG in North America, New Zealand, and Australia completed a study in low risk patients (COG P9641) using the INSS stage, age, *MYCN* status, INPC, and tumor cell ploidy to determine eligibility with a goal of showing that surgery alone was sufficient in low risk patients with stage 2 disease [36]. During the same time, the International Society of Pediatric Oncology Europe Neuroblastoma (SIOPEN) group completed a low risk study, SIOPEN LNESG1, that required patients to have surgically resectable localized tumors that were *MYCN* nonamplified [37]. This study also wanted to show that all patients with localized resectable disease, except those with stage 2 *MYCN* amplified tumors, could be successfully treated with surgery alone. The German Society of Pediatric Oncology and Hematology (GPOH) also conducted trials (GPOH NB95-S and NB97) during this period evaluating only infants less than 12 months of age with localized *MYCN* nonamplified tumors and looking at the ability to treat without cytotoxic chemotherapy [38]. In these low risk studies all completed between 1995 and 2004, only COG utilized histology and tumor cell ploidy. Additionally, only COG included patients with INSS stage 4S disease with this group of low risk patients. COG did not exclude patients with *MYCN* amplified tumors in the case of stage 1 tumors or infants under 12 months with stage 2 tumors, or older patients with stage 2 tumors with favorable histology by INPC. In the low risk patients, all groups looked at eliminating chemotherapy unless a patient was either symptomatic or had high risk features such as *MYCN* amplification.

Several intermediate risk studies were completed between 1997 and 2006 as well. COG A3961, a study conducted through COG utilized certain factors similar to the low risk study including age, *MYCN* status, INSS stage, INPC, and tumor cell ploidy to evaluate the ability to maintain excellent outcomes in patients with intermediate risk disease with reduced intensity chemotherapy [39]. Infants under 365 days with *MYCN* nonamplified, INSS stage 3 and 4 disease or 4s disease with either unfavorable histology per INPC or diploid tumors were included. Older patients were included with *MYCN* nonamplified INSS stage 3 disease with favorable histology by INPC. In Europe, SIOPEN completed several studies to evaluate these intermediate risk patients. SIOPEN 99.1 enrolled infants less than 12 months with localized, unresectable *MYCN* nonamplified tumors and the ability to utilize low dose chemotherapy and surgery alone [40]. SIOPEN 99.2 enrolled infants with *MYCN* nonamplified, metastatic disease that did not have metastases to the bone, central nervous system, lung, or pleura to evaluate the ability to only give chemotherapy for symptomatic patients [41]. In parallel, SIOPEN 99.3 enrolled infants with *MYCN* non-amplified disease who were not eligible for 99.2. This study evaluated the ability to give low dose chemotherapy in this cohort. SIOPEN EUNS evaluated patients older than 12 months with *MYCN* nonamplified localized, unresectable tumors and the ability to give low dose chemotherapy in these patients [42]. Similar to the low risk studies, only COG included tumor cell ploidy and histology. While there is likely overlap between the SIOPEN and COG intermediate risk cohorts, it is unclear if the populations are truly comparable due to the differences in criteria used to determine risk. Each of these intermediate risk studies evaluated the ability to give low dose chemotherapy without sacrificing the good outcomes expected in this group.

Among high-risk trials, inclusion criteria also varied among cooperative groups. In the United Kingdom, the Children’s Cancer and Leukemia Group completed CCLG-NB-1990-11 enrolling all patients over 12 months with metastatic disease to evaluate if a shortened interval between chemotherapy cycles will lead to improved survival [43]. SIOPEN enrolled patients with Evans stage 3 or 4 disease responsive to induction chemotherapy on ENSG1 to evaluate the role of high dose chemotherapy in this population [44]. The CCG completed CCG 3891 to evaluate the role of autologous stem cell transplantation versus chemotherapy for consolidation followed by 13-cis-retionoic acid for the treatment of high-risk neuroblastoma. This study utilized age, INSS stage, *MYCN* status, INPC histology, and ferritin in risk classification and study inclusion criteria [45]. They included patients over 12 months with INSS stage 4 disease, INSS stage 3 disease with either *MYCN* amplification, elevated ferritin, or unfavorable histology, INSS stage 2 disease with *MYCN* amplification, or INSS stage 1 or 2 disease that recurs after initial resection. GPOH completed a study (GPOH NB97) looking at patients over 12 months with INSS stage 4 disease and patients with *MYCN* amplified tumors of other stages comparing outcomes with autologous stem cell transplant versus maintenance chemotherapy [46]. After the CCG study, COG completed COG A3973 using age, *MYCN* status, INSS stage, and INPC including patients under 12 months with *MYCN* amplified INSS stage 3, 4, or 4s disease, patients over 12 months with INSS stage 4 or stage 3 disease with *MYCN* amplification or unfavorable histology or stage 2 disease with unfavorable histology and *MYCN* amplification, as well as patients who originally had low stage disease that returned with metastatic disease to determine whether purging neuroblastoma cells from autologous stem cells improves outcomes [47]. Finally, SIOPEN completed a SIOPEN HR-NBL1 evaluating patients over 12 months with INSS stage 4 disease and patients with stage 2, 3, or 4 disease with *MYCN* amplification to evaluate the need for IL-2 in addition to dinutuximab during maintenance therapy [48]. In general, CCG and then COG included additional features beyond what the various European groups utilized. All groups incorporated stage, age, and *MYCN* status, while CCG and COG also utilized ferritin and histology via INPC as in the lower risk groups.

**Figure 2:**
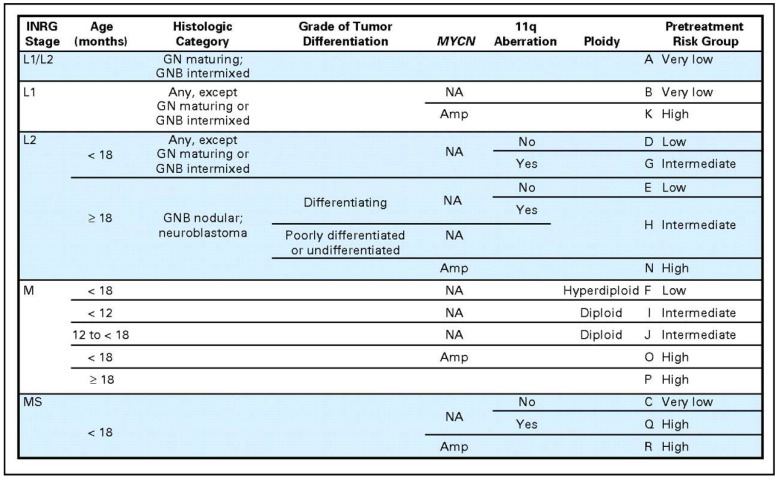
International Neuroblastoma Risk Group (INRG) classification system [4]. Reprinted with permission from American Society of Clinical Oncology © 2009, The International Neuroblastoma Risk Group (INRG) Classification System: An INRG Task Force Report; published by American Society of Clinical Oncology, 2009.

In order to address this problem of non-uniformly defined risk groups, a taskforce was assembled to develop the International Neuroblastoma Risk Group (INRG) classification system [4]. First, a new staging system was established that was based on image-defined risk factors of the original tumor rather than degree of surgical resection and allows for pretreatment risk stratification [13,49]. With the new staging system defined, a new risk stratification system was created as well. This was done by analyzing data collected on 8800 patients diagnosed between 1990 and 2002 in North America, Australia, Europe, and Japan. From this cohort, the most predictive factors were identified including INRG stage, age, histologic category, grade of differentiation, *MYCN* status, 11q aberration, and tumor cell ploidy. These features were used to define 17 cohorts that were categorized as very low, low, intermediate, or high risk. Patients categorized as very low risk had 5-year EFS of >85%, low from >75% to ≤85%, intermediate from >50 to <75%, and high <50% [4]. These groups were put into a risk stratification table that could be adopted to standardize the risk stratification of neuroblastoma patients (Figure 2).

As more data becomes available, the INRG risk classification system will continue to evolve. With this, it will be possible to better identify and define very low-risk or very high risk, or “ultra-high-risk” subgroups and therapy can be tailored accordingly. Morgenstern et al. [50] was able to identify a very high-risk subset of SIOPEN patients from the SIOPEN HR-NBL1 study using age, LDH, and number of metastatic sites. Saarinen-Pihkala et al. [51] identified an ultra-high-risk cohort as well using *MYCN* amplification and presence of bone metastases at diagnosis. Using the INRG database, a larger group of patients could be used to further investigate the definition of very or ultra-high-risk. Also with ongoing research into better ways to refine risk-stratificaiton, there will be novel factors added to the risk stratification schema. For instance, *TERT* rearrangements and *ATRX* mutations lead to alterations in telomere maintenance and are associated with more aggressive disease [52,53]. Circulating tumor DNA is another area of active research being done to identify less invasive way to characterize and monitor tumors [54]. These or other similar findings can be added to the risk stratification schema to better subdivide patients and assign the most appropriate treatment regimens. 

## 4. Conclusions

This INRG risk stratification system has not yet been adopted by all of the cooperative groups, but it represents the first step in being able to perform cross-cooperative group studies in order to optimize therapy. The INRG has already served to increase collaboration and data sharing between cooperative groups. Additionally, as technology and our understanding of the underlying biology of neuroblastoma improve, genomic features beyond *MYCN* amplification, ploidy, and segmental chromosome aberrations will likely be incorporated in risk classification. Additional refinement of risk classification will allow patients to receive the ideal therapy for his/her tumor in order to optimize outcomes but limit exposure to treatment-related toxicities. 

## Figures and Tables

**Table 1:** International Neuroblastoma Staging System (INSS).

INSS Stage	Description
1	Localized tumor, grossly resected, no lymph node involvement
2A	Unilateral tumor, incomplete gross excision, negative lymph nodes
2B	Unilateral tumor with positive ipsilateral lymph nodes
3	Tumor infiltrating across midline or unilateral tumor with contralateral lymph nodes or midline tumor with bilateral lymph nodes
4	Distant metastatic disease
4S	Localized primary tumor as defined by stage 1 or 2 in patient under 12 months with dissemination limited to the liver, skin, and/or bone marrow (<10% involvement)

**Table 2:** International Neuroblastoma Risk Group Staging Sysem (INRGSS).

INRG Stage	Description
L1	Localized tumor with no image-defined risk factors [13]
L2	Localized tumor with one or more image-defined risk factors [13]
M	Distant metastatic disease
MS	Metastatic disease in children under 18 months with metastases limited to skin, liver, and/or bone marrow (<10% involvement)

**Table 3:** International Neuroblastoma Pathology Classificaiton (INPC) histology definitions.

Favorable Histology	Unfavorable Histology
Ganglioneuroma mature (stroma-dominant)	Ganglioneuroblastoma, nodular (composite; stroma-rich/stroma-dominat and stroma-poor)
Ganglioneuroma maturing (stroma-dominant)	Neuroblastoma (stroma-poor)—all else not in favorable histology category
Ganglioneuroblastoma, intermixed (stroma-rich)	
Neuroblastoma (stroma-poor), differentiating or poorly differentiated with low/intermediate MKI in patients <1.5 years at diagnosis	
Neuroblastoma (stroma-poor), differentiating with low MKI in patients 1.5–5 years at diagnosis	

**Table 4:** Studies performed and the factors used for risk stratification.

Risk Group	Study	Factors Used for Risk Stratification
Low	COG P9641	INSS stage, age, *MYCN* status, INPC, tumor ploidy
Low	SIOPEN LNESG1	Surgically resectable localized tumor, *MYCN* status
Low	GPOH NB95-S and NB97	Age, localized tumor, *MYCN* status
Intermediate	COG A3961	INSS stage, age, *MYCN* status, INPC, tumor ploidy
Intermediate	SIOPEN 99.1	Age, localized unresectable tumors, *MYCN* status
Intermediate	SIOPEN 99.2	Age, metastatic disease to certain locations, *MYCN* status
Intermediate	SIOPEN EUNS	Age, localized unresectable tumors, *MYCN* status
High	CCLG-NB-1990-11	Age, metastatic disease
High	SIOPEN ENSG1	Evans stage, response to induction chemotherapy
High	CCG 3891	INSS stage, age, *MYCN* status, INPC, histology
High	GPOH NB97	Age, INSS stage, *MYCN* status
High	COG A3973	INSS stage, age, *MYCN* status, INPC
High	SIOPEN HR-NBL1	INSS stage, age, *MYCN* status
